# Proteomic study of facial melasma^⋆^

**DOI:** 10.1016/j.abd.2021.06.010

**Published:** 2022-09-10

**Authors:** Luiza Vasconcelos Schaefer, Leticia Gomes de Pontes, Nayara Rodrigues Vieira Cavassan, Lucilene Delazari dos Santos, Hélio Amante Miot

**Affiliations:** a Department of Pathology, Faculty of Medicine, Universidade Estadual Paulista, Botucatu, SP, Brazil; b Department of Dermatology, Universidade do Oeste Paulista, São Paulo, SP, Brazil; c Department of Research, Centro de Estudos de Venenos e Animais Peçonhentos, Universidade Estadual Paulista, Botucatu, SP, Brazil; d Postgraduate Program in Tropical Diseases, Faculty of Medicine, Universidade Estadual Paulista, Botucatu, SP, Brazil; e Department of Dermatology, Faculty of Medicine, Universidade Estadual Paulista, Botucatu, SP, Brazil

Dear Editor,

Melasma is hypermelanosis that affects photoexposed areas, especially in adult women, with a significant impact on quality of life by affecting visible areas and being recurrent, despite treatments. Its pathophysiology is not yet fully understood, but it results from the interaction between exposure factors (*e.g*., solar radiation and sex hormones) and genetic predisposition. Several dermal stimuli have been identified in the maintenance of melanogenesis in melasma, including the activity of fibroblasts, endothelium and mast cells, which promote elastonization of collagen, structural damage to the basement membrane, the release of growth factors (*e.g*., sSCF, bFGF, NGF, HGF) and inflammatory mediators (*e.g*., ET1, IL1, VEGF, TGFb).^1^, ^2^, ^3^

This study aimed to explore differentially exposed proteins in melasma skin when compared to adjacent, unaffected, photoexposed skin.

A cross-sectional study was carried out involving 20 women with facial melasma, without specific treatments for 30 days. Two biopsies were performed (by the same researcher), one at the edge of facial melasma and another on unaffected skin, 2 cm away from the first, as previously standardized.^1^, ^3^ The mechanical extraction of proteins was performed, followed by their enzymatic digestion and mass spectrometry. The project was approved by the institutional ethics committee (n. 1,411,931).

The samples were analyzed in duplicate in the nanoACQUITY-UPLC system coupled to a Xevo-Q-TOF-G2 mass spectrometer, and the results were processed with the ProteinLynx Global Server 3.03v software. The proteins were identified using the ion-counting algorithm, whose spectral patterns were searched in the *Homo sapiens* database, in the UniProt catalog (https://www.uniprot.org/).

All identified proteins with >95% similarity were included in the analysis. The intensities of the ion peaks were normalized, scaled and compared between topographies by a Bayesian algorithm (Monte Carlo method), which returns a value of p ≤ 0.05 for down-regulated proteins and ≥0.95 for up-regulated proteins, corrected by the Benjamini-Hochberg procedure.^4^

The main outcome of the study was the difference between the intensities of the ionic peaks of the proteins (Melasma: M, Perilesional: P). The effect size was estimated by the ratio of these amounts between topographies (M/P). Proteins with an M/P ratio of ≤0.5 or ≥2.0 were considered in this study.

The identified proteins and their biological functions were diagrammed in a heat map and grouped using the cluster procedure (Ward method).

The mean age (standard deviation) of the patients was 42.8 (8.9) years old, 70% were phototypes III‒IV and 25% worked in professions in which they were exposed to the sun. The age of melasma onset was 29.3 (7.5) years; 55% of the women reported a family history and 30% used contraceptives.

A total of 256 proteins were validated in the skin samples, and the 29 proteins differentially quantified between the topographies are shown in Table 1. The greatest discrepancies occurred for proteins HBD, EXPH5, KRT1, KRT9, REV3L (M/S > 4,00); and ACAP9, ADGB, CA1 (M/S < 0.33).

**Table 1:** Proteins and isoforms identified in samples of facial melasma (M) and adjacent photoexposed (P) skin (n = 40) with the difference between the groups (p ≤ 0.05 or ≥0.95) and M/P ratio ≥2.0 or ≤0.5.

Protein code	Protein	PLGS score	Melasma	Perilesional	Log2 M/P (sd)	M/P ratio	p-Value^a^
P1	Actin Alpha Skeletal Muscle ACTA1	958.81	1.34 (0.04)	0.66 (0.04)	1.04 (0.07)	2.05	1.00
P2	Actin Cytoplasmic 2 ACTG1	1164.96	1.40 (0.06)	0.60 (0.06)	1.23 (0.11)	2.34	1.00
	Actin Cytoplasmic 2 ACTG1	1164.96	1.40 (0.07)	0.60 (0.07)	1.24 (0.12)	2.36	1.00
P3	A-Kinase Anchor Protein 13 AKAP13	87.85	1.53 (0.40)	0.47 (0.40)	1.96 (1.03)	3.90	0.97
	A-Kinase Anchor Protein 13 AKAP13	87.85	1.55 (0.37)	0.45 (0.37)	1.99 (1.13)	3.97	0.95
P4	A-kinase Anchor protein 9 AKAP9	12.43	0.35 (0.34)	1.65 (0.34)	−2.39 (1.00)	0.19	0.03
	A-kinase Anchor protein 9 AKAP9	13.08	0.38 (0.20)	1.62 (0.20)	−2.16 (0.50)	0.22	0.00
	A-kinase Anchor protein 9 AKAP9	16.30	0.40 (0.24)	1.60 (0.24)	−2.06 (0.55)	0.24	0.00
P5	Albumin isoform CRA k ALB	542.99	0.62 (0.05)	1.38 (0.05)	−1.14 (0.08)	0.45	0.00
	Serum albumin ALB	5862.44	0.54 (0.18)	1.46 (0.18)	−1.44 (0.32)	0.37	0.00
	Serum albumin ALB	542.99	0.63 (0.04)	1.37 (0.04)	−1.14 (0.07)	0.45	0.00
	Serum albumin ALB	542.99	0.52 (0.07)	1.48 (0.07)	−1.50 (0.12)	0.35	0.00
	Serum albumin ALB	2923.53	0.62 (0.10)	1.38 (0.10)	−1.17 (0.17)	0.44	0.00
	Serum albumin ALB	542.99	0.63 (0.06)	1.37 (0.06)	−1.11 (0.10)	0.46	0.00
	Serum albumin ALB	486.61	0.64 (0.06)	1.36 (0.06)	−1.07 (0.09)	0.48	0.00
	Serum albumin ALB	534.91	0.66 (0.08)	1.34 (0.08)	−1.04 (0.12)	0.49	0.00
P6	Alpha-1-antitrypsin SERPINA1	1092.60	1.33 (0.13)	0.67 (0.13)	1.00 (0.21)	2.00	1.00
P7	Androglobin ADGB	69.19	0.48 (0.21)	1.52 (0.21)	−1.69 (0.40)	0.31	0.05
P8	Annexin ANXA2	117.73	1.33 (0.25)	0.67 (0.25)	1.01 (0.41)	2.01	0.95
	Annexin ANXA2	117.73	1.33 (0.26)	0.67 (0.26)	1.02 (0.44)	2.03	0.97
	Annexin ANXA2	117.73	1.34 (0.26)	0.66 (0.26)	1.05 (0.43)	2.08	0.95
	Annexin ANXA2	117.73	1.35 (0.28)	0.65 (0.28)	1.08 (0.43)	2.12	0.95
P9	Beta-actin-like protein 2 ACTBL2	101.00	1.43 (0.07)	0.57 (0.07)	1.34 (0.12)	2.53	1.00
P10	BTB/POZ domain-containing protein KCTD7	53.81	1.57 (0.17)	0.43 (0.17)	1.90 (0.40)	3.74	1.00
P11	Carbonic Anhydrase 1 CA1	1112.39	0.39 (0.17)	1.61 (0.17)	−2.09 (0.45)	0.23	0.00
	Carbonic Anhydrase 1 CA1	1386.45	0.47 (0.13)	1.53 (0.13)	−1.70 (0.27)	0.31	0.00
P12	Ceruloplasmin CP	76.55	1.40 (0.17)	0.60 (0.17)	1.23 (0.30)	2.34	1.00
	Ceruloplasmin CP	85.85	1.37 (0.17)	0.63 (0.17)	1.13 (0.29)	2.18	1.00
	Ceruloplasmin CP	85.85	1.43 (0.15)	0.57 (0.15)	1.34 (0.27)	2.53	1.00
P13	DNA polymerase zeta catalytic subunit REV3L	73.85	1.58 (0.34)	0.42 (0.34)	2.03 (0.86)	4.10	0.95
	DNA polymerase zeta catalytic subunit REV3L	153.25	1.52 (0.06)	0.48 (0.06)	1.66 (0.12)	3.16	1.00
P14	Exophilin-5 EXPH5	40.12	1.79 (0.12)	0.21 (0.12)	3.16 (0.48)	8.94	1.00
P15	Fibrinogen Gamma chain FGG	211.06	1.33 (0.16)	0.67 (0.16)	1.01 (0.25)	2.01	1.00
P16	Fibrinogen Gamma chain FGG	211.06	1.34 (0.14)	0.66 (0.14)	1.04 (0.24)	2.05	1.00
	Fructose-bisphosphate Aldolase A ALDOA	153.06	1.46 (0.07)	0.54 (0.07)	1.43 (0.14)	2.69	1.00
	Fructose-bisphosphate aldolase A ALDOA	296.85	1.45 (0.07)	0.55 (0.07)	1.41 (0.13)	2.66	1.00
	Fructose-bisphosphate aldolase ALDOA	295.30	1.46 (0.10)	0.54 (0.10)	1.43 (0.17)	2.69	1.00
	Fructose-bisphosphate aldolase ALDOA	295.30	1.47 (0.08)	0.53 (0.08)	1.46 (0.14)	2.75	1.00
P17	G Patch domain-containing protein 1 GPATCH1	95.48	1.37 (0.14)	0.63 (0.14)	1.13 (0.25)	2.18	1.00
	G patch domain-containing protein 1 GPATCH1	88.85	1.60 (0.28)	0.40 (0.28)	2.15 (0.66)	4.44	1.00
P18	Heat shock protein 75 kDa mitochondrial TRAP1	124.78	1.43 (0.13)	0.57 (0.13)	1.34 (0.24)	2.53	1.00
P19	Hemoglobin subunit alpha HBA1	8552.23	1.57 (0.02)	0.43 (0.02)	1.88 (0.04)	3.67	1.00
P20	Hemoglobin subunit beta HBB	91.85	0.65 (0.05)	1.35 (0.05)	−1.07 (0.08)	0.48	0.00
P21	Hemoglobin subunit delta HBD	42.06	1.94 (0.02)	0.06 (0.02)	5.05 (0.31)	33.12	1.00
P22	Keratin type I cytoskeletal 9 KRT9	340.12	1.61 (0.13)	0.39 (0.13)	2.05 (0.27)	4.14	1.00
	Keratin type I cytoskeletal 9 KRT9	190.36	1.60 (0.26)	0.40 (0.26)	2.06 (0.62)	4.18	1.00
P23	Keratin type II cytoskeletal 1 KRT1	55.74	1.62 (0.06)	0.38 (0.06)	2.08 (0.14)	4.22	1.00
P24	POTE ankyrin domain family member F POTEF	101.00	1.47 (0.07)	0.53 (0.07)	1.49 (0.14)	2.80	1.00
P25	Putative beta-actin-like protein 3 POTEKP	101.00	1.47 (0.09)	0.53 (0.09)	1.47 (0.16)	2.77	1.00
P26	RNA-binding protein 25 RBM25	29.17	1.57 (0.12)	0.43 (0.12)	1.86 (0.25)	3.63	1.00
P27	Splicing Regulatory glutamine/Lysine-rich protein 1 SREK1	79.89	1.41 (0.26)	0.59 (0.26)	1.28 (0.48)	2.44	1.00
P28	Tetratricopeptide repeat protein 37 TTC37	443.53	1.45 (0.21)	0.55 (0.21)	1.44 (0.42)	2.72	1.00
	Tetratricopeptide repeat protein 37 TTC37	449.05	1.46 (0.18)	0.54 (0.18)	1.47 (0.36)	2.77	1.00
P29	Triosephosphate isomerase TPI1	475.34	1.39 (0.25)	0.61 (0.25)	1.23 (0.41)	2.34	0.97
	Triosephosphate isomerase TPI1	475.34	1.41 (0.22)	0.59 (0.22)	1.28 (0.40)	2.44	0.97
	Triosephosphate isomerase TPI1	576.90	1.43 (0.19)	0.57 (0.19)	1.34 (0.37)	2.53	1.00

a p-Value corrected by false discovery rate.

The main biological functions of these proteins are shown in Table 2. Fig. 1 represents the interaction between the 29 proteins and their biological functions. Proteins ACTG1, ALB, SERPINA1, HBD, ALDOA, and FGG showed to be co-participants in different biological processes, such as oxygen consumption, glycolysis, gluconeogenesis, and cell transport, suggesting an increase in the metabolic activity of the skin with melasma.

**Table 2:** Main functional pathways associated with the 29 proteins identified as differentials between melasma and perilesional skin.

Functions	Involved proteins	n (%)	FDR^a^
1. Canonical glycolysis	p16, p29	2 (7)	<0.0001
2. Gluconeogenesis	p16, p29	2 (7)	<0.0001
3. Fibrinolysis	p8, p15, p23	2 (10)	<0.0001
4. Platelet degranulation	p2, p5, p6, p15, p16	5 (17)	<0.0001
5. Regulation of body fluids	p2, p5, p6, p8, p15, p16, p21, p22, p23	8 (28)	<0.0001
6. Oxygen transport	p7, p19, p20, p21	4 (14)	<0.0001
7. Vesicle-mediated transport	p2, p5, p6, p10, p14, p15, p16, p19, p20	9 (31)	<0.0001
8. Platelet activation	p5, p6, p15, p16	4 (14)	<0.0001
9. Positive regulation of cell adhesion	p15	1 (3)	0.0001
10. Hemostasis	p2, p5, p6, p15, p16, p21	6 (20)	0.0001
11. Platelet aggregation	p2, p15	2 (7)	0.0002
12. Plasminogen activation	p15	1 (3)	0.0003
13. Single-organism transport	p2, p5, p6, p7, p8, p10, p11, p12, p14, p15, p16, p19, p20, p21	14 (48)	0.0004
14. Blood clotting	p2, p5, p6, p15, p16, p21	6 (21)	0.0008
15. Error-prone translesion synthesis	p13	1 (3)	0.0010
16. Protein activation cascade	p15, p23	2 (7)	0.0014
17. Retinal homeostasis	p2, p5, p23, p24	4 (14)	0.0015
18. Down-regulation of trauma response	p8, p10, p15	3 (10)	0.0015
19. Up-regulation of exocytosis	p14, p15	2 (7)	0.0016
20. Regulation of exocytosis	p10, p14, p15	3 (10)	0.0017
21. Down-regulation of endothelial cell apoptosis process	p15	1 (3)	0.0022
22. Blood clotting, fibrin clot formation	p15	1 (3)	0.0024
23. Down-regulation of the extrinsic apoptosis signaling pathway through the receptor death domain	p15	1 (3)	0.0024
24. Transport	p2, p4, p5, p6, p7, p10, p11, p12, p15, p16, p19, p20, p21	13 (45)	0.0029
25. Monocarboxylic Acid Metabolic Process	p5, p16, p29	3 (10)	0.0030
26. Regulation of adhesion-dependent cell spread	p15	1 (3)	0.0033
27. Wound healing	p2, p5, p6, p15, p16, p21	6 (20)	0.0035
28. Bicarbonate transport	p11, p19, p20	3 (10)	0.0044
29. Up-regulation of vasoconstriction	p15	1 (3)	0.0044
30. Response to calcium ion	p2	1 (3)	0.0075
31. Regulation of transport by vesicles	p8, p10, p14, p15	4 (14)	0.0083
32. Secretion	p5, p6, p8, p15, p16	5 (17)	0.0092
33. Down-regulation by external stimuli	p8, p10, p15	3 (10)	0.0098
34. Response to stress	p2, p5, p6, p13, p16, p19, p20, p21, p23	9 (31)	0.0100

a False discovery rate estimated according to the number of proteins expected for the function.

**Figure 1:**
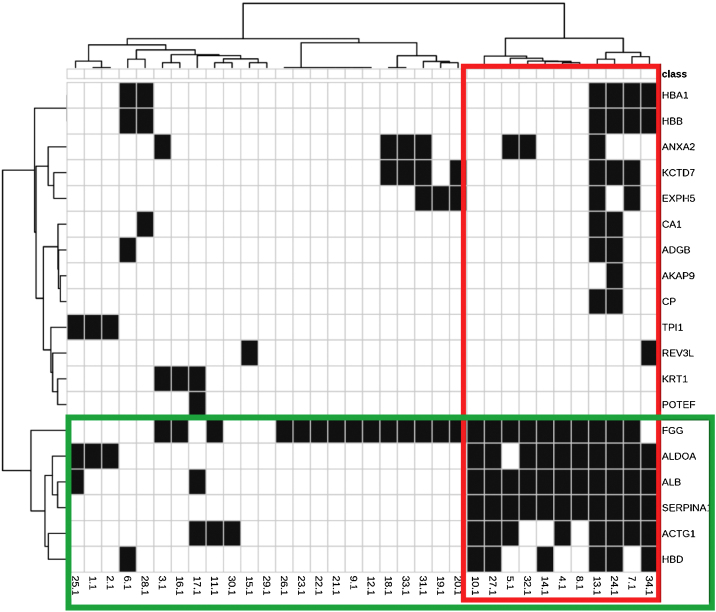
Heat map and dendrograms between identified proteins (rows) and biological functions (columns). Green highlights: grouping of proteins with a similar pattern of occurrence according to the functions they perform; and in red: the functions with a similar expression pattern, according to the indicated proteins.

Exophyllin-5 (EXPH5) is linked to intracellular vesicle transport. It was up-regulated (M/S = 8.94) in melasma, which may be due to the intense epidermal transfer of melanosomes.^1^ Thirteen of the proteins differentially identified in melasma have been linked to intracellular transport phenomena, which comprise a series of processes ranging from endocytosis to autophagy and several forms of exocytosis. As autophagy and senescence are melanogenesis-related phenomena, characterization of transport vesicles in the melasma epithelium may prove to be important in the pathophysiology of melasma.^5^, ^6^

Cytokeratins (such as KRT1) are structural constituents of keratinocytes induced in response to oxidative stress. They were identified in greater proportion in melasma (M/S > 4.10). Hemoglobin-δ (but not the other subunits) showed a high ratio (M/S = 33.12) in melasma, and, in addition to oxygen transport, its non-erythrocytic expression occurs in situations of cell stress.^7^ Likewise, up-regulation of alpha 1-antitrypsin (SERPINA1) and actin gamma-1 (ACTG1) is also seen in tissue stress conditions.^8^, ^9^ The higher expressions of HBD, ACTG1, SERPINA1, and KRT1 in melasma may be due to oxidative stress sustained by mast cell tryptase activity and the secretory phenotype of upper dermis fibroblasts.^3^, ^6^

Carbonic anhydrase (CA1) acidifies the extracellular environment of the dermis, favoring the repair process, being down-regulated (M/S < 0.33) in melasma.^10^ The senescence of dermal fibroblasts, associated with the activity of MMP1 and MMP9, promotes a pro-inflammatory microenvironment with degradation of the extracellular matrix and the basement membrane zone, the repair deficit of which may be a factor in the maintenance of melanogenesis.^1^, ^6^

Androglobin (ADGB) has a cysteine-endopeptidase regulatory function, being identified in a lower ratio (M/S < 0.33) in melasma. Endopeptidases participate in the degradation of melanosomes in the epidermis, notably reduced in melasma.

The alpha-kinase anchor proteins (ANCHOR9, ANCHOR13) and the z-catalytic subunit of DNA polymerase (REV3L) showed an imbalance in the skin with melasma. They are important in the regulation of protein kinase-A and the p38-MAP kinase pathway, involved in the activation of the CREB protein, which leads to the expression of MTIF, a promoter of melanogenesis.^3^

Aldolase-A (ALDOA) ​​has a glycolytic function and is associated with the activity of mast cells, which, in the superficial dermis, promote changes in the basement membrane, solar elastosis, and endothelial dilation, reinforcing the idea that stimuli originating in the dermis play a central role in the melanogenesis of melasma.^2^, ^3^

Fibrinogen-γ (FFG) is an extracellular matrix protein, and interacts in several biological functions, including fibrinolysis, fibrinogen activation and activation of the ERK pathway, a promoter of melanogenesis.

The main limitations of the study are related to transmembrane, serum and lipid-conjugated proteins, which are not identified by the method. However, it consistently points to a number of proteins with a pathophysiological role and potential therapeutic manipulation of which should be explored in specific assays.

In conclusion, the study identified 29 differentially regulated proteins in melasma, involved in energy metabolism, cell transport phenomena, regulation of melanogenesis pathways, hemostasis/coagulation, repair/healing, and response to oxidative stress. This supports the research of therapeutic strategies aimed at the identified proteins and their functions and shows that melasma does not depend exclusively on the hyperfunction of melanocytes but also on functional alterations involving the epidermal melanin unit, basement membrane zone and upper dermis.

## Financial support

FUNADERSP (048/2016).

## Authors’ contributions

Luiza Vasconcelos Schaefer: Design and planning of the study; drafting and editing of the manuscript; collection, analysis, and interpretation of data; intellectual participation in the propaedeutic and/or therapeutic conduct of the studied cases; critical review of the literature.

Leticia Gomes de Pontes: Collection, analysis, and interpretation of data.

Nayara Rodrigues Vieira Cavassan: Collection, analysis, and interpretation of data.

Lucilene Delazari dos Santos: Critical review of the literature; critical review of the manuscript; collection, analysis, and interpretation of data.

Hélio Amante Miot: Critical review of the literature; critical review of the manuscript; statistical analysis; approval of the final version of the manuscript; design and planning of the study.

## Conflicts of interest

None declared.
